# Optimisation of Mechanical Properties of Gradient Zr–C Coatings

**DOI:** 10.3390/ma14020296

**Published:** 2021-01-08

**Authors:** Łukasz Szparaga, Przemysław Bartosik, Adam Gilewicz, Katarzyna Mydłowska, Jerzy Ratajski

**Affiliations:** Faculty of Mechanical Engineering, Koszalin University of Technology (KUT), Śniadeckich 2, 75-453 Koszalin, Poland; bartosikprzemyslaw@o2.pl (P.B.); adam.gilewicz@tu.koszalin.pl (A.G.); katarzyna.mydlowska@tu.koszalin.pl (K.M.); jerzy.ratajski@tu.koszalin.pl (J.R.)

**Keywords:** gradient coatings, optimisation, anti-wear coatings, FEM modeling, functionally graded materials

## Abstract

One of the key components of the designing procedure of a structure of hard anti-wear coatings deposited via Physical Vapour Deposition (PVD) is the analysis of the stress and strain distributions in the substrate/coating systems, initiated during the deposition process and by external mechanical loads. Knowledge of residual stress development is crucial due to their significant influence on the mechanical and tribological properties of such layer systems. The main goal of the work is to find the optimal functionally graded material (FGM) coating’s structure, composed of three functional layers: (1) adhesive layer, providing high adhesion of the coating to the substrate, (2) gradient load support and crack deflection layer, improving hardness and enhancing fracture toughness, (3) wear-resistant top layer, reducing wear. In the optimisation procedure of the coating’s structure, seven decision criteria basing on the state of residual stresses and strains in the substrate/coating system were proposed. Using finite element simulations and postulated criteria, the thickness and composition gradients of the transition layer in FGM coating were determined. In order to verify the proposed optimisation procedure, Zr-C coatings with different spatial distribution of carbon concentration were produced by the Reactive Magnetron Sputtering PVD (RMS PVD) method and their anti-wear properties were assessed by scratch test and ball-on-disc tribological test.

## 1. Introduction

Currently, hard anti-wear coatings, mainly multi-layer, composed of nitrides, carbides, oxides and borides of transition metals Ti, Cr, Zr, W, Mo, V, Ta, Nb, Hf [1] are widely deposited by Physical Vapour Deposition (PVD) on, among others, parts of the machines and mechanisms as well as cutting and forming tools for metal and wood [2,3,4]. However, the complexity of the structure and composition of multilayer coatings causes problems primarily related to the mutual adhesion of the individual layers. This mainly affects layers with radically different mechanical properties, e.g., TaC/TiC/Al_2_O_3_/TiN [5]. Moreover, in this type of coating, discontinuities of stress and deformation (step change in values of tensor components) occur at the boundaries of the layers, which significantly increases the probability of the formation of cohesive cracks and local delamination at the interfaces [5]. 

One of the possibilities of solving the above-mentioned problems occurring in multilayer coatings is the use of the concept of functionally graded materials (FGM) [6], characterised by a gradient of structure and chemical composition [4,7,8,9]. In particular, the analysis of residual stress states in single-layer (homogeneous), gradient and multilayer coating of the same thickness, resulting from external mechanical loads, show that among the three analyzed coatings, the maximum values of von Mises stresses and shear stresses are the lowest in a gradient coating. It has also been shown that in gradient coatings, under external stresses, the location of the maximum shear stresses shifts from the substrate/coating interface towards the upper zone of the coating, as the coating thickness increases in relation to the contact radius [10]. In addition, in the case of gradient coatings under mechanical loads (Hertzian contact), a smaller zone of high Tresci stresses is also observed in the area of the coating/substrate interface compared to homogeneous coatings. This results in greater resistance to the initiation and propagation of potential cracks in the area of the coating’s interface with the substrate [11].

Overall, the durability and reliability of a hard anti-wear coating require high adhesion to the substrate W_1_, low abrasive wear W_2_ and high fracture toughness W_3_. Hence, one of the important problems connected with the design of the coating’s structure in the case of multilayer coatings is the selection of the chemical composition, mechanical properties and thickness of individual layers and, in the case of FGM coatings, the selection of spatial distribution of its chemical composition, in order to achieve the maximum values of W_1_–W_3_ [12,13,14,15,16,17]. In this field, numerical simulations of the state of residual stresses and strains in the substrate-coating systems, arising after the deposition process, play a special role due to the significant impact of residual stresses on the mechanical and tribological properties of the coatings [18,19,20,21,22,23,24,25]. The analysis of stresses and deformations initiated as a result of external mechanical loads provides additional information about potential wear of coatings in various tribological tests, including ball-on-disc or pin-on-plate tests [26,27,28,29] and resistance to cracking in the scratch tests [30,31,32]. 

In the area of the above-mentioned issues, the work in [33] deserves special attention. In this work, a proposal was made to select the spatial distribution of Young’s modulus, which would enhance the gradient coating’s crack resistance, based on the stress distribution initiated in the coating by the so-called circular and elliptical Hertzian mechanical loads. The condition for selecting the optimal Young’s modulus profile was to minimize the maximum value of von Mises stresses on the interfaces and to reduce the tensile stresses in the coating. The research described in [34] was focused on the selection of the optimal Young’s modulus profile in the a-C gradient coating, due to the so-called contact damages in indentation tests, based on experimental tests and Finite Element Method (FEM) simulations. It has been shown that the maximum value in the spatial distribution of Young’s modulus should be in the half of the coating thickness, which results in a significant reduction in the size of potential cracks at higher indentation depths. Therefore, the obtained results clearly document that, in order to improve the fracture toughness of thin PVD gradient coatings, there should be a region where monotonicity changes in the optimal Young’s modulus profile.

In summary, despite the significant progress made in the design of gradient anti-wear coatings, no generally acceptable and unambiguous set of optimisation guidelines and criteria has been formulated to date. Hence, this work is part of the search for representative criteria correlated with their anti-wear properties in order to develop an optimisation procedure. The present work is a continuation of the research presented by the authors in [35] on the optimisation of the structure of a multilayer coating in terms of its wear resistance. In the optimisation procedure presented by the authors in [35], a set of six decision criteria was adopted, the values of which were calculated on the basis of FEM numerical simulations of the stress and strain state in the coating under external loads. The internal stresses in the layers (growth stresses + thermal stresses) initiated in the deposition process were assumed as the initial stress state in the FEM model. Using a proposed procedure, a number of multi-layer coatings prototypes were selected and grouped into subsets which included prototypes with potentially similar mechanical properties. In order to verify the proposed optimisation procedure, Zr-C coatings with different carbon concentrations in each layer were produced by the RMS PVD method and their anti-wear properties were assessed by scratch test, Rockwell C indentation tests and ball-on-disc tribological test. It was shown that prototypes with low values of decision criteria are characterised by higher critical loads in scratch test, and by lower wear ratios.

The main goal of the work is to find an optimal FGM coating’s structure, composed of three functional layers: (1) adhesive layer, providing high adhesion of the coating to the substrate, (2) gradient load support and crack deflection layer, improving hardness and enhancing fracture toughness, (3) wear-resistant top layer, reducing wear. The procedure adopts seven decision criteria, which are functions of stresses and strains in the FGM coating induced by external tangential and normal mechanical loads. Among the newly proposed criteria, there are also three previously developed and tested criteria [35] adapted for the current optimisation procedure of FGM coatings. Using an optimisation procedure with the postulated criteria, an optimal FGM coating’s structure was proposed. Verification of the proposed optimisation procedure was carried out on the Zr-C coatings deposited by RMS PVD. As shown in [36,37,38,39], the change in carbon concentration in Zr-C coatings allows for the shaping of their mechanical properties (hardness, yield strength, Young’s modulus, and residual stresses) in a wide range, which has become the criterion for selecting these coatings for verification of optimisation procedure. The choice of Zr-C coatings was also dictated by their wide field of application, including cutting and forming tools and as components of microelectromechanical devices [36]. Selected prototypes of coatings were prepared and then subjected to experimental tests aimed at assessing their anti-wear properties.

## 2. Materials and Methods

### 2.1. Optimisation

#### 2.1.1. Object

**Figure 1:**
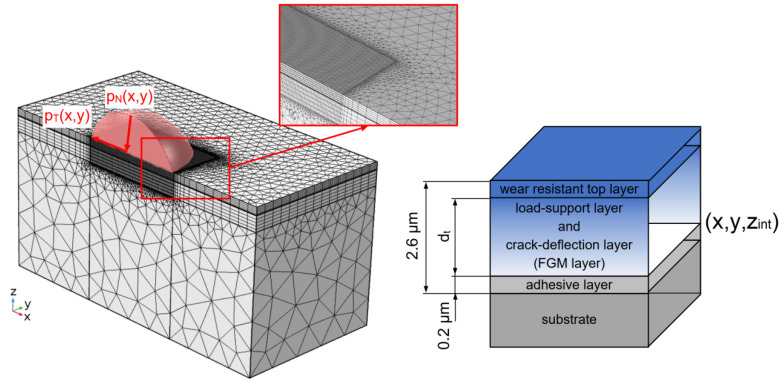
General structure of optimisation object (functionally graded material (FGM) coating) with mesh grid.

The optimisation object is a gradient coating composed of three functional layers: adhesive layer, gradient load support and crack deflection layer, and wear-resistant top layer, with a structure as in Figure 1, deposited on an HS-6-5-2 steel substrate. HS-6-5-2 (DIN-1.3343) steel is a high-speed steel with the following composition, mass concentration of elements (wt.%): C (0.82–0.92), Mn (max 0.4), Si (max. 0.50), P (max. 0.03), S (max. 0.03), Cr (3.5–4.5), W (6–7), Mo (4.5–5.5), V (1.7–2.1), Cu (max. 0.3) and Fe—balanced. Zr–C coatings were chosen as functional layers, with different carbon concentration gradients, deposited via RMS PVD. 

Two decision variables were postulated in the optimisation task: (1) thickness of gradient load support and crack deflection layer (*d_t_*), (2) exponent of the power transition function describing the continuous change in carbon concentration in the gradient layer (*p*). The domain of decision variables is defined as follows

(1) $$\left[d_{t},p\right]\in D=\left\{1,\;1.25,\;1.5,\;1.75\;,2.0\right\}\;µm\times \left\{\frac{1}{4},\frac{1}{3},\frac{1}{2},1,2,3,4\right\}$$

**Figure 2:**
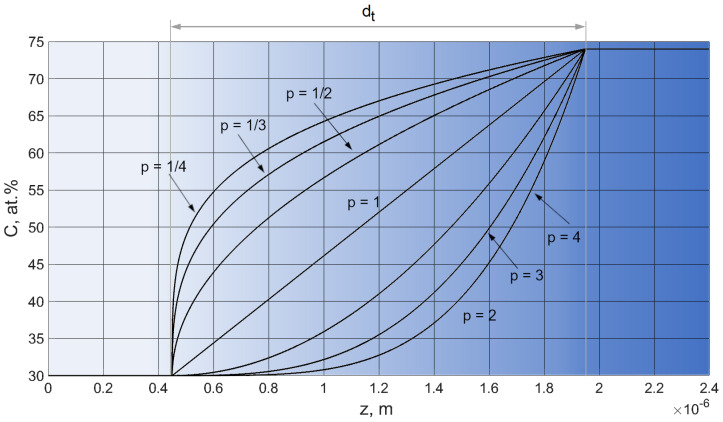
Carbon concentration profiles for analysed set of *p* parameters.

The explicit formula of the adopted power transition functions, describing the spatial distribution of carbon concentration in the gradient layer, is given by

(2) $$C=\left\{\begin{array}{c} C_{ZrC\left(base\right)},\;\;z\in \left[0\;;0.5d_{total}-0.5d_{t}\right]\\ C_{ZrC\left(base\right)}+\left(C_{ZrC\left(top\right)}-C_{ZrC\left(base\right)}\right){\left(\frac{z-0.5d_{total}+0,5d_{t}}{d_{t}}\right)}^{p},\;z\in \left[0.5d_{total}-0.5d_{t}\;;0.5d_{total}+0.5d_{t}\right]\\ C_{ZrC\left(top\right)},\;\;z\in \left[0.5d_{total}+0.5d_{t};\;d_{total}\right] \end{array}\right.,$$

where, respectively, *C_ZrC_*_(*base*)_ and *C_ZrC_*_(*top*)_ denote the carbon concentration at the boundaries of the adhesive layer/gradient layer and gradient layer/top layer, *d_total_*—total coating thickness, *d_t_*—gradient layer thickness. The form of the transition function defined by Equation (2) makes it possible to identify the change in the exponent *p* with the change in the carbon concentration in the coating. Figure 2 shows the carbon concentration profiles for the analysed set of parameters (*p*), for fixed thickness of gradient layer (*d_t_*). 

In the optimisation procedure, the coating’s thickness was assumed constant, equal to 2.6 µm. Based on the authors’ previous experimental and theoretical investigations [37,38,39], as the adhesive layer was assumed to be a Zr metallic layer with thickness 0.2 µm [37,38,39]. The use of this Zr metallic layer between a steel substrate and a hard Zr-C layer significantly improves the adhesion of the coating to the substrate by, among others, a reduction in the residual stresses in the interfaces generated by differences in lattice parameters and coefficient of thermal expansion (CTE) of the substrate and coatings. In the FEM model of the substrate/coating system, the following assumptions and simplifications were made:

- The steel substrate is treated as a homogeneous continuous medium;
- Substrate and layer materials are elastic-plastic bodies represented by a so-called bi-linear model with work hardening;
- Before deposition process in substrate initial stresses were 0 GPa;
- At the boundary between the substrate and the coating mesh nodes are connected—no separation allowed;
- Spatial distribution of carbon concentration in gradient layer is represented by a continuous power transition function;
- A homogeneous temperature distribution was assumed in the samples during the deposition of the coatings—the deposition temperature value was 400 °C.

The COMSOL Multiphysics 5.2a program (Structural Mechanics and Nonlinear Structural Materials packages) [40] was used to create a three-dimensional FEM model of the substrate with the deposited coating. 

**Figure 3:**
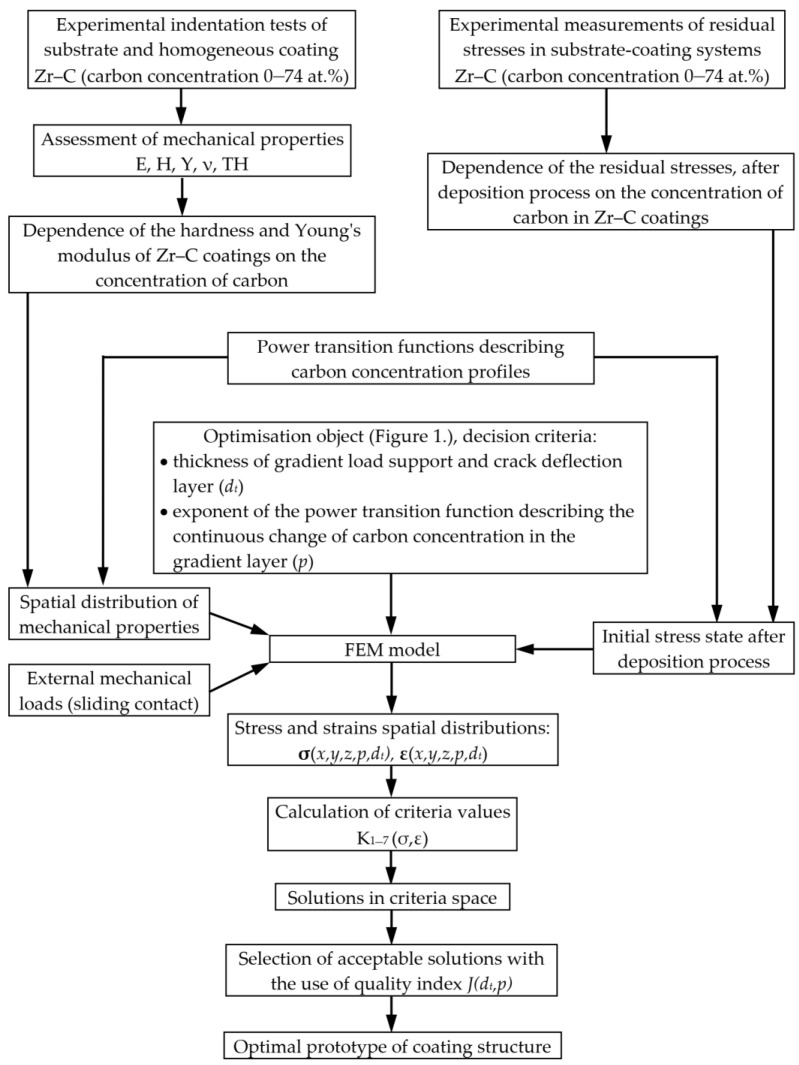
Scheme of the optimisation procedure.

The aim of the research undertaken was to find the carbon concentration profile in FGM layer (load support and crack deflection layer) and its thickness, which would provide high coating fracture toughness, adhesion and wear resistance. A scheme of the proposed optimisation procedure is presented in Figure 3.

#### 2.1.2. Decision Criteria

**Figure 4:**
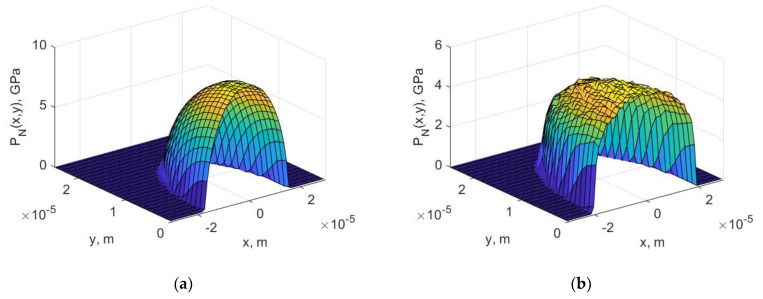
Distribution of normal contact pressure on the surface of the top layer initiated by a spherical indenter with a radius of 200 µm (**a**) elastic contact, (**b**) elasto-plastic contact.

Basing on classical Hertzian elastic contact theory [41], in sliding contact, e.g., rigid ball sliding against coated sample, normal $p_{N}\left(x,y\right)$ and tangential $p_{T}\left(x,y\right)$ loads acting on top surface are given by

(3) $$p_{N}\left(x,y\right)=P_{0}{\left[1-{\left(\frac{x}{a}\right)}^{2}-{\left(\frac{y}{a}\right)}^{2}\right]}^{0.5},\;\;p_{T}\left(x,y\right)=\mu \cdot p_{N}\left(x,y\right),$$

where *P*_0_—maximum contact pressure, *µ*—coefficient of friction, *a*—radius of contact. The maximum value of the contact pressure and the contact radius determined for the elastic deformation range differ significantly when taking into account the substrate’s plastic deformation. Namely, in the case of taking into account the plastic deformations of the substrate and the coating, the contact area increases and the maximum value of the contact pressure decreases compared to those obtained for pure elastic contact [42,43,44]. Exemplary distributions of normal contact pressure $p_{N}\left(x,y\right)$ on the surface of the top layer, initiated by a spherical indenter with a radius of 200 µm, obtained on the basis of FEM simulation, are shown in Figure 4a elastic contact, Figure 4b elasto-plastic contact. In this paper, for the purposes of optimisation in further calculations, only the $p_{N}\left(x,y\right)$ distribution obtained from elasto-plastic contact will be used (Figure 4b).

Optimisation of carbon concentration profile in FGM layer (load support and crack deflection layer) and its thickness was done for given continuous 3D external mechanical loads—quasi-static equivalent of the scratch test with diamond indenter with a radius of 200 µm. In the optimisation procedure, seven decision criteria based on the state of stresses and strains in the coating, resulting from internal stresses (after deposition process) and imposed external mechanical loads, was proposed. The first prototype of the set of decision criteria proposed in this paper was presented by the authors in [35]. One of the main differences from the optimisation criteria presented in [35] for multilayer coatings (2D FEM simulations) is the fact that, currently, each of the adopted criteria is determined on the basis of a 3-dimensional state of stress and strain (3D FEM simulations). Moreover, three new decisional criteria were added to the set of previously used criteria.

The *K*_1_ criterion was the weighted sum of the fraction of positive first-principal stresses *σ_I_* and their average value in a given volume of the coating. The explicit formula of this criterion is

(4) $$K_{1}\left(d_{t},p\right)=weight\cdot \sigma_{I\left(pos\right)}+\left(1-weight\right)\cdot \sigma_{I\left(ave\right)},$$

(5) $$f\left(i\right)=\left\{\begin{array}{c} 0,\;\sigma_{I}\left(i\right)\leq \;0\\ 1,\sigma_{I}\left(i\right)>\;0\; \end{array}\right.,\;\;n=\sum_{i=1}^{N}f\left(i\right),\;\;\;\sigma_{I\left(pos\right)}=\frac{n}{N},\;\;\;\sigma_{I\left(ave\right)}=\frac{\sum_{i=1}^{N}f\left(i\right)\cdot \sigma_{I}\left(i\right)}{n},$$

where *i* is the element number and volume *N* denotes total number of elements in mesh grid (in the analysed coating’s volume).

As *K*_2_ criterion was adopted the maximum value of *I* principal stress in the zone of interface of adhesive layer/gradient layer (Figure 1).

(6) $$K_{2}\left(d_{t},p\right)=max\left(\sigma_{I}\right),$$

The *K*_1_ and *K*_2_ criteria were already tested as potential measures of coating’s resistance to cracking (through thickness perpendicular cracks) and the mechanical integrity of the adhesive layer/gradient layer interface [35,45,46].

Criterion *K*_3_ was the maximum value of equivalent plastic deformations in the adhesive layer

(7) $$K_{3}\left(d_{t},p\right)=max\left(\varepsilon_{eqv}\right),$$

As shown in [27,28,29,35], this criterion is well correlated with potential resistance to gradual frictional wear.

Criterion *K*_4_ was the maximum value of the module of shear stresses *σ_xy_* in the adhesive layer/gradient layer interface (Figure 1)

(8) $$K_{4}\left(d_{t},p\right)=max\left|\left(\sigma_{xy}\left(x,y,z_{int}\right)\right)\right|,$$

The choice of this criterion is dictated by the relationship between the shear stresses at the layer boundaries and the resistance to the initiation of lateral cracks, which may lead to coating delamination [45,46,47]. Research results presented in [48,49] document that compressive stresses are responsible for buckling at any poorly adhered zone of the coating, developed in the region ahead of the indenter in scratch test. This fact was a premise to define the *K*_5_ criterion as the maximum value of III principal stress in the coating.

(9) $$K_{5}\left(d_{t},p\right)=max\left(\left|\;\sigma_{III}\right|\right),$$

Therefore, according to the interpretation shown in [49], the value of *K*_5_ could be a significant indicator connected with the probability of coating buckling.

The mean value of von Mises stresses in the coating was adopted as the *K*_6_ criterion

(10) $$K_{6}\left(d_{t},p\right)=\;\sigma_{vm\left(ave\right)}=\frac{\sum_{i=1}^{N}\sigma_{vm}\left(i\right)}{N},$$

where *i* is the element number and *N* is the total number of elements in the analysed coating’s volume in FEM model. The inspiration for introducing this criterion was the work of [50], in which the relationship between coating wear and the distribution of stresses in coatings, initiated in contact with a spherical indenter, was investigated. The results presented in this work clearly document the relationship between the mean value of von Mises stresses in the coating’s volume and the value of the wear index determined in the pin-on-plate test for CoAu coatings.

The maximum value of the *σ_zy_* stresses on the adhesive layer/gradient layer interface was adopted as the criterion *K*_7_.

(11) $$K_{7}\left(d_{t},p\right)=max\left|\left(\sigma_{zy}\left(x,y,z_{int}\right)\right)\right|,$$

The *K*_7_ criterion was introduced due to the relationship of shear stresses in the plane perpendicular to the direction of the indenter motion, and the probability of chipping and delamination due to recovery spallation after the disappearance of the external load at the indenter-coating contact boundary [32,45,46,48]. The proposed set of decision criteria is a synthesis of various relationships, known in the literature, between the stress and strain distributions in the coatings with the probability of occurrence inter alia: coating’s spallation, conformal and angular cracking, brittle tensile cracking. According to the definitions of the introduced criteria, their simultaneous minimisation is required for providing enhanced resistance for the occurrence of coating’s failure mode, thus optimal coating prototypes should be characterised by low values of the adopted criteria. The scaling of the adopted decision criteria to a dimensionless form significantly facilitates the analysis of the set of obtained solutions in the criteria space. Thus, criteria were normalised as follows

(12) $$K_{i}^{\left(n\right)}=\frac{K_{i}-K_{i}^{min}}{K_{i}^{max}-K_{i}^{min}}i=1,\ldots ,7K_{i}^{\left(n\right)}\in \left[0,1\right],$$

*K_i_^min^* and *K_i_^max^* denote, respectively, the minimal and maximal value of criteria in the defined domain (1). In the further presentation of the results, only normalized criteria will be analysed without upper index (*n*), i.e., *K_i_*^(*n*)^ ≡ *K_i_*.

### 2.2. Experimental Details

#### 2.2.1. Coatings Deposition

Zr–C gradient coatings were deposited via reactive magnetron sputtering (RMS) on substrates made of HS5-6-2 steel, in the form of discs that are 32 mm in diameter and 3 mm thick as well as rectangular silicon substrates with dimensions 10 × 20 × 0.5 mm. The steel samples were quenched (preliminary annealing ~550 °C, >30 min, austenitization ~1050 °C, 20 min, cooling in oil) and double tempering at 540 °C. The samples in the quenched and tempered condition with a hardness of approx. 63 HRC were provided by FABA S.A. Prior to deposition, the substrates were ground and polished, which resulted in obtaining a mirror state with roughness parameters on average R_a_ = 0.023 µm, R_z_ = 0.187 µm. (R_a_—Arithmetical mean deviation of the assessed profile, R_z_—Maximum roughness height according to ten profile points). Grinding was carried out with a semi-automatic grinder with water-cooled SiC grain sandpaper. The papers were changed gradually from granulation 100 to 2000. The surfaces were then polished with pastes with a monocrystalline diamond suspension with an average grain size of 3 µm, then 1 µm, on a polishing cloth of acetate fibers. Before the deposition process, the samples were washed with acetone at 58.08 g/mol and rinsed in an ultrasonic cleaner with EcoShine Ultrasonic K3 solution containing <5% of each individual of the three nonionic surfactants sodium 5–15 edetate (EDTA), <5% corrosion inhibitor then they were rinsed in deionized water and dried. In the working chamber, substrates were subjected to ion cleaning at a pulse voltage of a glow discharge with an amplitude of 1.5 kV at an Ar pressure of 7 Pa. Sputtering was performed from a metallic Zr target, with a diameter of 114 mm supplied by PLANSEE Composite Materials, Lechbruck, Austria. The target was made by powder metallurgy. Composition: Zr + Hf min. 99.2 wt.%, Hf max. 4.5 wt.%, Fe + Cr max. 2000 µg/g, H max. 50 µg/g, N max 250 µg/g, C max. 500 µg/g, O max. 1600 µg/g, at fixed discharge power of 500 W in a mixed Ar and C_2_H_2_ atmosphere by controlling the flow rate of both gases. Different profiles of carbon concentrations in the coatings were obtained by changing the flow rate of C_2_H_2_ in the range of 1.5–6.5 sccm. The magnetron source was powered with pulse voltage with a frequency of 1 kHz with a signal modulated by 100 kHz. Substrates were polarised with negative 10 V bias voltage, and their temperature during all the deposition processes was stabilised in the range of 400 ± 20 °C.

#### 2.2.2. Characterisation Methods

After depositing the Zr-C coatings on silicon substrates, the value of the residual stresses was estimated by measuring the surface profiles of the samples (determining the radius of curvature) with a Hommelwerke T2000 profilometer (Hommelwerke GmbH, Villingen-Schwenningen, Germany) using the Stoney formula.

Indentation measurements were made using a Fisherscope HM2000 (Fischer, Sindelfingen, Germany) micro hardness tester equipped with a Berkovich diamond indenter and WIN-HCU^®^ 4.3 software. To determine the value of Young’s modulus and hardness using the Olivier–Pharr model [51], indentation tests were carried out with a given maximum force and constant penetration rate, obtaining load vs. depth curves.

The fracture toughness and adhesion of the coatings were assessed by scratch test using a CSM Revetest scratch tester (CSM Instruments SA, Needham, MA, USA). The tester was equipped with a diamond conical indenter with a spherical tip of 200 µm radius. The indenter was moved across the surface of the coating at a speed of 10 mm/min; the force loading the indenter increased linearly from the preload of 90 mN at a speed of 100 N/min to 50 N over a distance of 5 mm. During the test, the normal and tangential force were recorded as a function of the indenter displacement, the acoustic emission signal was recorded and the resulting scratch path was simultaneously observed using optical microscope. On this basis, the value of the load at which the first coating damage occurred (called the critical load L_c1_) was determined, as well as the load value at which the coating was completely removed from scratch path (L_c3_).

Tribological tests were carried out using the ball-on-disc method with the use of a T-10 Tester device (ITEE, Radom, Poland), measuring the force and friction coefficient as a function of the sliding distance. The tests were carried out with a normal load of 5 N and with a linear sliding speed of 0.2 m/s. A ceramic alumina ball with a diameter of 10 mm was used as a counter-specimen. Based on the cross-section profiles of the wear tracks obtained with the Hommelwerke T2000 profilometer (Hommelwerke GmbH, Villingen-Schwenningen, Germany), the values of the volumetric wear ratio of the sample were calculated.

## 3. Results and Discussion

### 3.1. Simulation Results

#### 3.1.1. Criteria Values in the Decision Variables Domain

**Figure 5:**
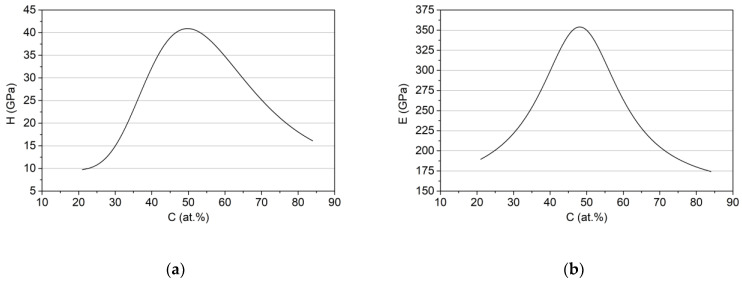
Dependence of the hardness (**a**) and Young’s modulus; (**b**) of Zr–C coatings on the concentration of carbon.

The determination of the values of basic material constants of Zr-C coatings, depending on the carbon concentration, i.e., Young’s modulus E, yield strength Y, tangent modulus TM, as well as the initial residual stress σ_0_, is necessary to calculate the values of the adopted decision criteria, using the created FEM models, for each coating prototype in the domain of decision variables. For this purpose, single-layer Zr-C coatings with a carbon concentration of 21 at.%, 51 at.%, 66 at.%, 74 at.% and 84 at.% on HS-6-5-2 steel and Si substrates were produced. Details on the methodology of sample preparation and coating deposition conditions are included in the authors’ previous works [37,38,39]. Then, experimental indentation tests were performed and the values of Young’s modulus and hardness in the function of carbon concentration were determined (Figure 5a,b) using procedures discussed in Section 2.2.

**Figure 6:**
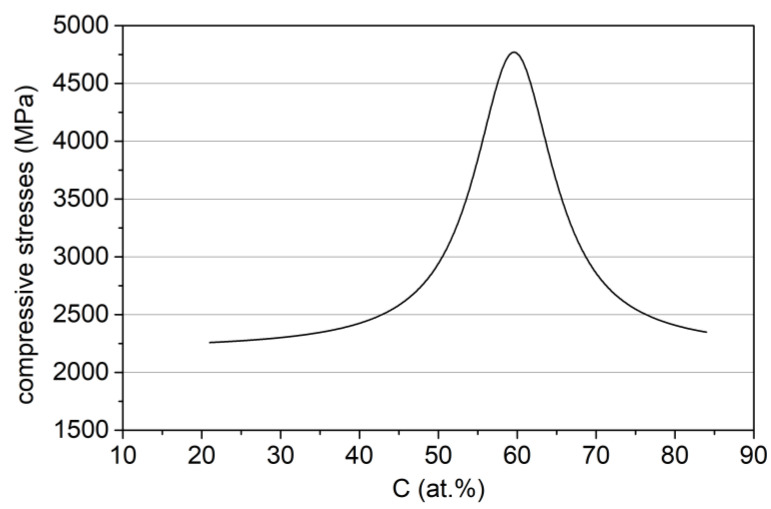
Dependence of the residual stresses, after deposition process, in Zr–C coatings on the concentration of carbon.

Yield strength Y and tangent modulus TM in the function of carbon concentration are assumed to be proportional, respectively, to H(C), E(C) functions (Figure 5a,b) which is a general rule for a wide class of materials [52,53,54]. The dependence between the mean value of the compressive stresses in the Zr–C coating in the (*x,y*) plane and the carbon concentration was also determined (Figure 6).

Residual stresses were assessed after the coating’s deposition process basing on measurements of the radius of curvature of the Si/Zr–C substrate/coating systems and calculated using Stoney’s formula [55].

**Figure 7:**
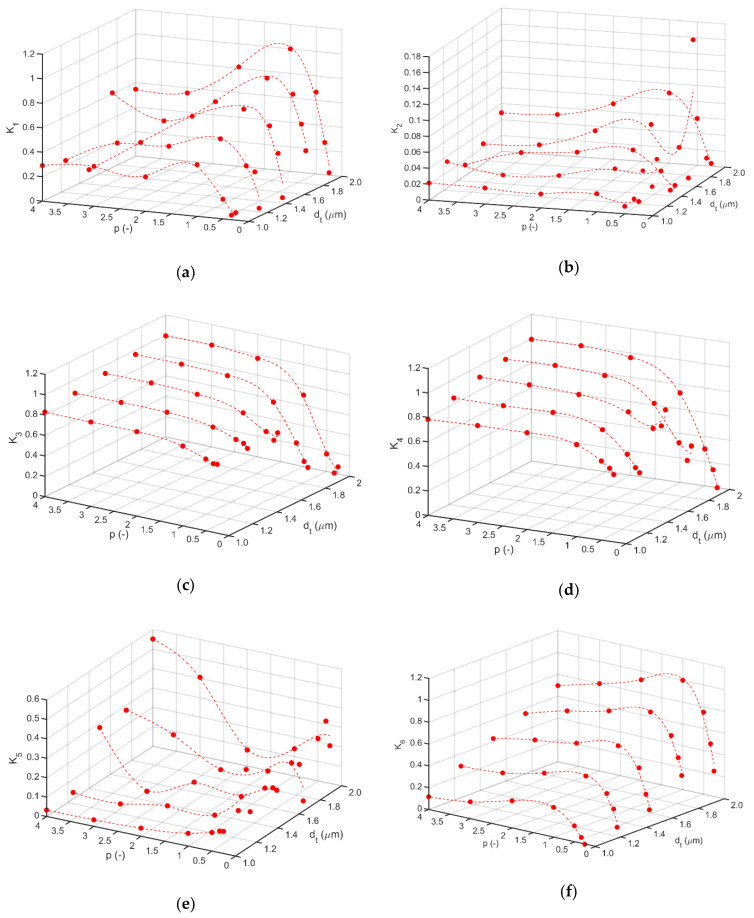

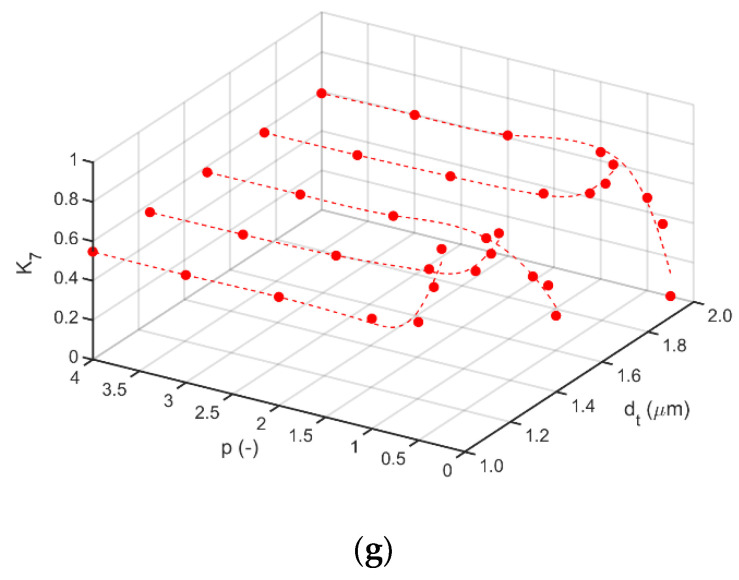
Dependences of *K*_1_–*K*_7_ (**a**–**g**) on values of parameter *p* and thickness of gradient layer (*d_t_*).

For the postulated domain of decision variables, (1) dependences of *K*_1_–*K*_7_ on values of parameter *p*, of transition function, and thickness of gradient layer (*d_t_*), were determined (Figure 7a–g).

According to the adopted definition and normalization of each of the criteria, the value 0 corresponds to the minimum (desirable), and the value 1 (unfavorable) to the global maximum. The obtained explicit dependencies *K_i_* = *K_i_*(*d_t_*,*p*), *i* = 1, 5, 6 indicate the presence of relatively large changes in the value of the criteria in the neighborhood of the decision variable *p* = 1 for each thickness *d_t_*. It follows that the prototype with *p* = 1 is a kind of a limit prototype in the neighborhood of which significant differences in mechanical properties of prototypes can be expected. It should be noted that in the case of the *K_i_* = *K_i_*(*d_t_*,*p*), *i* = 1, 7 (criteria related to the potential resistance to tensile cracks and chipping initiation), the increment in gradient layer thicknesses (*d_t_*) results in a strong differentiation of the prototypes due to their potential anti-wear properties.

#### 3.1.2. Coating’s Prototypes

In order to perform the selection of all analyzed prototypes, represented by the points *K* = (*K*_1_, *K*_2_, *K*_3_, *K*_4_, *K*_5_, *K*_6_, *K*_7_) in the 7-dimensional space of decision criteria, the distance *d* = *d*(*d_t_*,*p*) was determined for each of them from the so-called utopian solution represented by the origin of the coordinate system.

(13) $$d\left(d_{t},p\right)=\sqrt{\sum_{i=1}^{7}\;K_{i}^{2}\left(d_{t},p\right)\;},$$

Moreover, for each acceptable solution, the number of criteria *N* = *N*(*d_t_*,*p*), which values are below the level of 0.3, was determined, i.e.,

(14) $$n\left(i\right)=\left\{\begin{array}{c} 1,\;\;K_{i}\leq \;0.3\\ 0,\;K_{i}>\;0.3\; \end{array}\right.,\;\;N\left(d_{t},p\right)=\sum_{i=1}^{7}n\left(i\right)\;\;,$$

Using the dependencies *d* = *d*(*d_t_*,*p*) and *N* = *N*(*d_t_*,*p*) the quality index was defined in the form

(15) $$J\left(d_{t},p\right)=\;\frac{d\left(d_{t},p\right)}{N\left(d_{t},p\right)},$$

**Figure 8:**
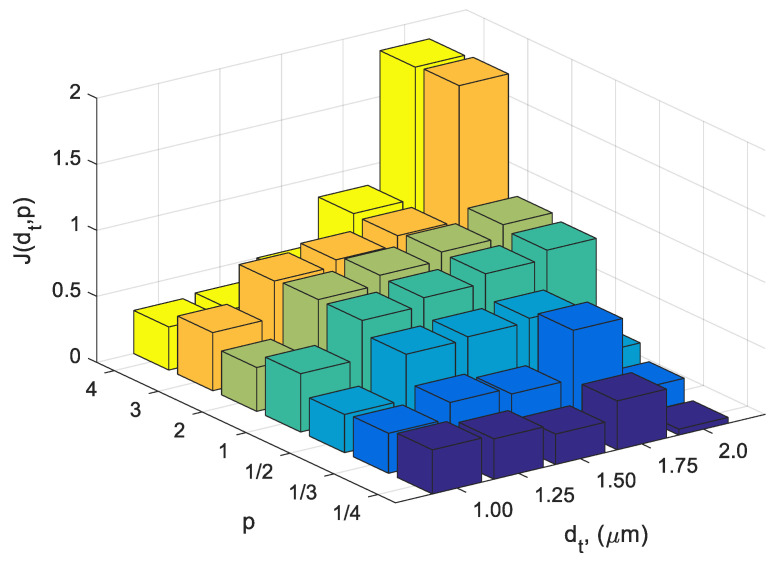
Quality criterion $J\left(d_{t},p\right)\;$ as a function of parameter (*p*) and thickness of gradient layer (*d_t_*).

In the case where the value of *N*(*d_t_*,*p*) was 0, the index *J*(*d_t_*,*p*) was not calculated. The introduced *J*(*d_t_*,*p*) quality index was the basic criterion for selecting the best solutions—coating prototypes. It contains information about the distance of the tested solution from the so-called utopian solution (the best possible theoretical acceptable solutions) in the criteria space and, at the same time, supports the selection of solutions for which individual criteria are below the value of 0.3. In further considerations, it was assumed that the level of 0.3 is a limit value below which the criterion, scaled to the fuzzy variable, is at the Low (L) level. The *J*(*d_t_*,*p*) index has been defined in such a way that its minimum value corresponds to the best coating prototype. For the analysed domain of decision variables (1), the chart of *J*(*d_t_*,*p*) is presented in Figure 8.

It should be noted that for gradient layers with the lowest thickness *d_t_* = 1 μm, the differences in the values of the criteria between the individual prototypes are low; therefore, these prototypes should have similar anti-wear properties. If the gradient layer reaches about 50% of the total thickness of the coating, an increase in the differences in the values of the criteria between individual prototypes is observed, which leads to their diversification in terms of potential anti-wear properties. Maximum differences in the values of the criteria are achieved in the case of a gradient layer with a thickness of *d_t_* = 2 μm, which is about 80% of the total thickness of the coating. Thus, in order to obtain the highest diversification of the prototypes in terms of their mechanical properties, prototypes with the highest gradient layer thickness were selected for further experimental research. In particular, prototypes were selected for which the index *J*(*d_t_*,*p*) has a minimal or maximal value (Table 1). They are potentially the best and the worst solutions in the considered domain of decision variables *D*. The table also includes a representative of the group of solutions, with the value of curvature parameter of the power transition function equal to *p* = 1, characterised by a linear increase in carbon concentration in the area of the gradient layer—limit prototype (Section 3.1.1).

**Figure 9:**
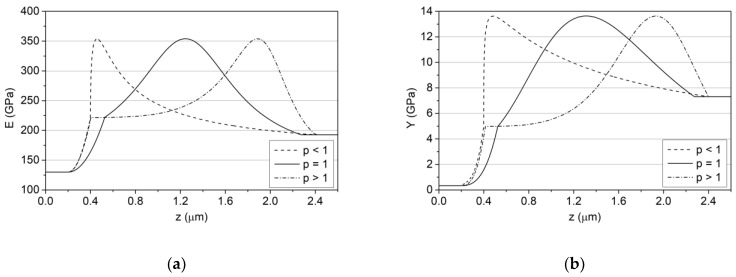
Spatial distributions of the Young’s modulus (**a**) and yield strength (**b**) for selected prototypes (*p* < 1, *p* = 1, *p* > 1).

By analysing Figure 8 and Table 1, it can be clearly stated that the prototypes characterised by *p* < 1 form a subset of solutions with lower values of the quality index *J*(*d_t_*,*p*) compared to prototypes with *p* > 1 for the same thickness of the *dt* gradient layer. Moreover, among the prototypes with *p* < 1, there is a prototype ensuring the global minimization of *J*(*d_t_*,*p*), while among the prototypes with *p* > 1, there is a prototype with the maximum value of this quality index. Hence, in further considerations, in order to draw general conclusions about the design of optimal carbon concentration profiles, the solutions will be divided into three groups together with their representatives (Table 1), i.e., *p* < 1; (*J*(*d_t_*,*p*)*→min*), *p* = 1 and *p* > 1; (*J*(*d_t_*,*p*)*→max*). Spatial distributions of the Young’s modulus and yield strength for selected prototypes are shown in Figure 9.

Then, selected coatings prototypes (Table 1) were produced, and their anti-wear properties were experimentally evaluated.

### 3.2. Experimental Results 

#### 3.2.1. Scratch Test

**Figure 10:**
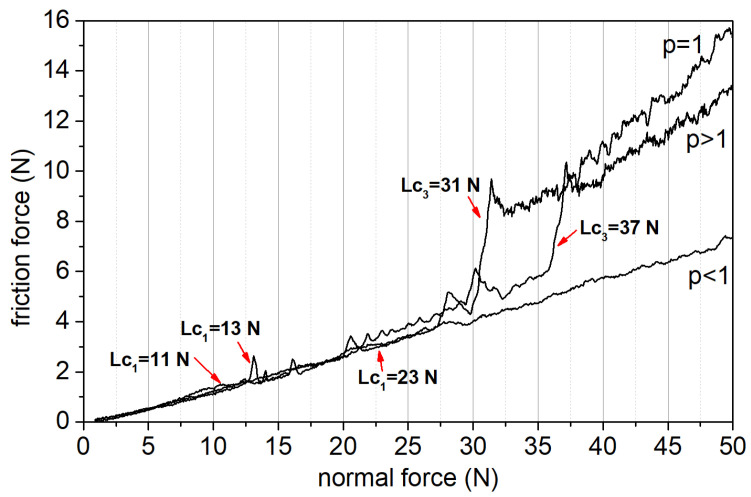
Frictional force versus normal force for selected prototypes—*p* > 1, *p* = 1, *p* < 1.

The fracture toughness and adhesion of the coatings, which are strongly connected with their anti-wear properties, were assessed by scratch test. Figure 10 shows the dependence of frictional force versus normal force for selected prototypes, determined during the test.

**Figure 11:**
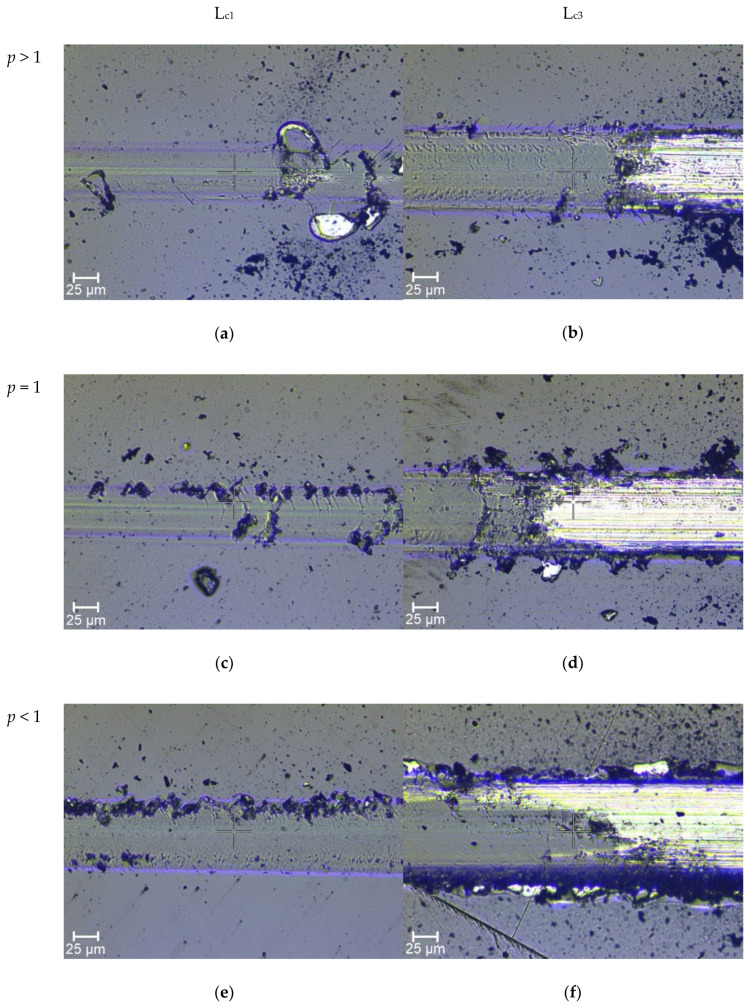
Microscopic photos of scratches for selected prototypes: *p* > 1 (**a**,**b**), *p* = 1 (**c**,**d**), *p* < 1, (**e**,**f**).

Microscopic photos of scratch path, illustrating damages of coatings for the L_c1_ and L_c3_ critical loads are presented in Figure 11a–f.

Damages occurring in the scratch path for the coating with the value of *p* > 1 (hardness H = 25 GPa, with a depth of 0.15 µm) are typical damages of hard brittle coatings (belonging to the group of brittle failure modes in scratch testing) deposited on high-hardness substrates, in this case, an HS -6-5-2 steel substrate with a hardness of H = 8 GPa. The first cracks in the coating (L_c1_ = 13 N), the so-called Chevron (angular) cracks, belong to the group of brittle tensile cracks. These cracks arise behind the indenter as a result of tensile stresses, whose values exceed coating tensile strength [56,57,58]. With these coatings, a further indenter load increment results in the compressive spallation, initiated in front of the indenter in the high-compressive-stress zone. It should also be emphasized that in the case of this prototype, there is a zone of high internal stresses (−4 to −4.7 GPa), directly under the top layer, which add up to the compressive stresses caused by the indenter, which additionally increases the rate of energy release and the size of initiated cracks. The coating’s complete delamination occurs for the value of L_c3_ = 37 N.

For the prototype with *p* = 1, damages in the scratch path belong to the group of ductile failure modes. The first cracks occurring in the coating (L_c1_ = 11 N), with few chippings and areas of delamination along the path, are so-called conformal cracks and are typical for relatively ductile coatings. They result from the action of compressive stresses in front of the indenter, which then slides over the fracture surface and pushes them into the path. With a further increase in the indenter load, buckling spallations become larger and spread to the sides of the scratch path. Consequently, this leads to the complete coatings detachment as the formed cracks extend over the entire thickness of the coating [56,57,58].

A prototype with *p* < 1 possesses the highest value of the critical force (L_c1_ = 23 N) among the tested prototypes. Coating damages in the scratch path, as in the case of the prototype with *p* = 1, belong to the ductile failure modes group. However, in the case of the prototype with *p* < 1, no buckling spallations or wedging are observed, which is characteristic of highly ductile coatings possessing relatively low hardness. For this prototype, only conformal cracks are observed, with a small amount of chipping along the scratch path until the coating is fully delaminated from the substrate. The critical force L_c3_, for this prototype is 60 N and is about two times higher than in other prototypes.

#### 3.2.2. Wear Test

**Figure 12:**
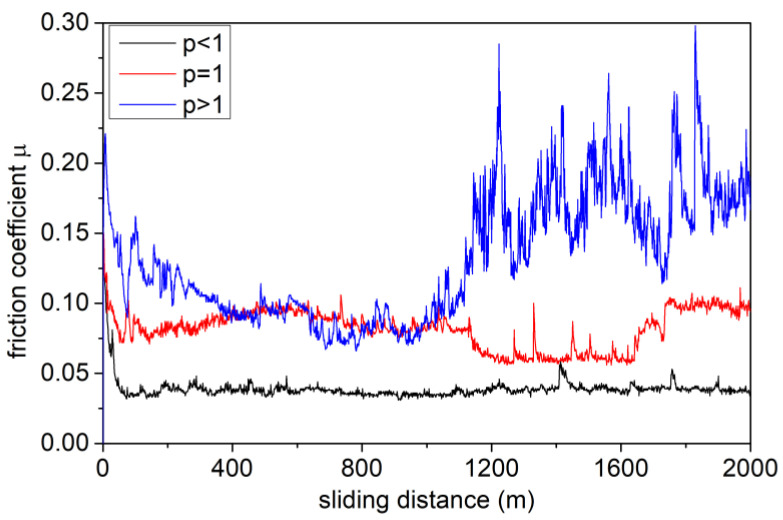
Coefficients of friction for selected prototypes vs. sliding distance.

For the purposes of wear assessment, for produced prototypes *p* > 1, *p* = 1 and *p* < 1, tribological tests (ball-on-disc) were performed, with simultaneous measurements of the frictional force vs. sliding distance. In the ball-on-disc wear tests, each coating prototype was made on six samples, then, on each of them, two measurements were performed (two measurements were made on each sample to avoid interactions between the obtained tracks and to achieve the same number of cycles at a given sliding distance). Figure 12 shows the dependence of the friction coefficient between the tested coating’s prototypes and the counter-sample vs. sliding distance (total measurement distance 2000 m).

In all investigated coating’s prototypes, the wear-resistant top layer had a carbon concentration ~74 at.%. However, for the *p* < 1 prototype, the friction coefficient is lower (COF = 0.045) than for the other prototypes (Figure 12). The differences in the COF values at the given normal force for individual prototypes result from the differences in the real contact areas with the alumina ball (counter-sample), which strongly depend on the mechanical properties of the coatings, e.g., [41,59]. Changes in the contact area cause changes in the value of the total frictional force, particularly its components: deformational, cohesive and adhesive. These individual components are responsible, respectively, for the initiation of: plastic deformation in the coating, wear products’ particle formation and surface blocking of the friction pair on irregularities [60]. It should be noted that, for prototypes *p* < 1 and *p* = 1, the dependence of the value of friction coefficient vs. sliding distance is relatively stable compared to the prototype with *p* > 1. This effect is related to the fact that in the case of the prototype with *p* > 1, directly below the top layer, there is a zone of high hardness with a carbon concentration of from 50 up to 60 at.%. Thus, as a result of contact with the counter-sample, hard-wear products appear, which cause ridging and scarfing of the surface and lead to the formation of microcracks. Energy-consuming processes, like breaking the cohesive bonds of the coating by the irregularities of the counter-sample and cyclic deformation of wear products in the frictional node, are also responsible for the higher value of the calculated COF for the prototype with *p* > 1 [60,61,62].

**Figure 13:**
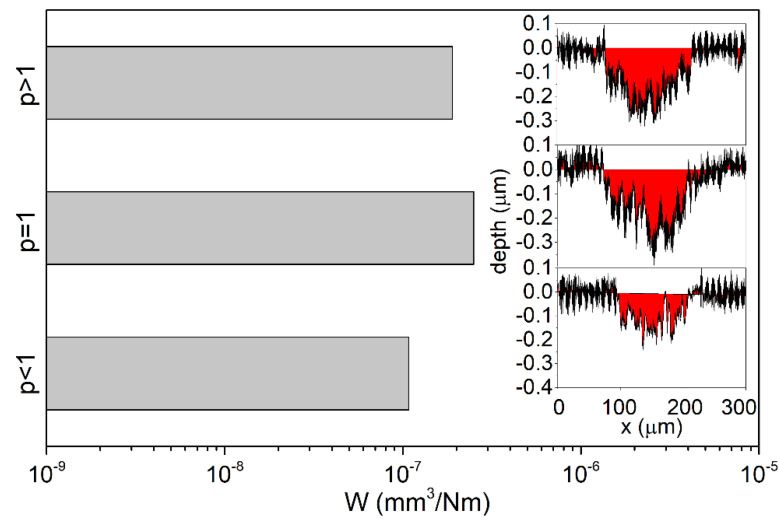
Wear ratio with cross-section of wear tracks for selected prototypes after ball-on-disc test, sliding distance 2000 m.

Using the cross profiles of the obtained wear track (Figure 13), the values of the volumetric wear ratio of the coating were calculated via the formula

(16) $$W=\frac{V}{P\cdot L},\;V=\frac{S_{1}+S_{2}+S_{3}}{3}\cdot 2\pi r,$$

where *P*—applied load in N, *L*-sliding distance in m and *V*-wear volume, *S*_1_, *S*_2_, *S*_3_—cross section of wear track, mm^2^, *r*—wear track radius, mm.

In all analysed prototypes, the wear-resistant top layer with a concentration of 74 at.% of carbon belongs to the group of diamond-like layers a-C:H:Zr in which the ZrC grains are in the matrix of amorphous carbon [39]. In the case of coatings characterised by a high content of a-C:H phase, a transfer layer with a graphitic structure, possessing very good lubricant properties, is formed during wear, which contributes to the uniform wear of the coating [61,62]. The lowest wear rate was observed for the prototype with *p* < 1, for which *W* = 1.08·10^−7^ mm^3^/N∙m was approx. two times lower compared to the prototypes with *p* = 1 and *p* > 1, for which the values of volumetric wear indicator were, respectively, *W* = 2.5·10^−7^ mm^3^/Nm and *W* = 1.9·10^−7^ mm^3^/Nm. The differences in the values of wear indicators are directly correlated with the obtained graphs of the values of COF vs. sliding distance (Figure 12) for the tested prototypes. Namely, the prototype with *p* < 1 characterised by the lowest volumetric wear ratio, possesses the lowest value of COF and relatively small fluctuations in its value vs. sliding distance.

### 3.3. Summary Results 

The aim of the conducted study was to optimise the gradient coating structure in terms the of anti-wear properties. The optimisation procedure developed as part of the research was based on the state of strains and stresses initiated by tangential and normal external mechanical loads, taking into account initial residual stresses in coatings. Two decision variables were adopted in the optimisation procedure: (1) thickness of gradient load support and crack deflection layer (*d_t_*), and (2) exponent of the power transition function, describing the continuous change in carbon concentration in the gradient layer. Seven decision criteria were adopted, being the functions of the stress and deformation states, which simultaneous minimisation allows for obtaining the optimal distribution of stress in terms of preventing the occurrence of cracks under a given load. In order to verify the proposed optimisation procedure, Zr-C coatings, with different spatial distributions of carbon concentration described by power functions, were produced. Table 2 presents the collective results of simulations and experimental tests for the analysed prototypes.

For the purposes of the analysis of the correlation of the adopted criteria with the obtained experimental results, the values of the *K*_1_–*K*_7_ criteria were rescaled to a fuzzy-logic variable in the form:

- $K_{1-7}\in \;\langle 0;0.1\rangle$—VL (Very Low);
- $K_{1-7}\in \;\left(0.1;\right.0.3\rangle$—L (Low);
- $K_{1-7}\in \;\left(0.3;\right.0.5\rangle$—M (Moderate);
- $K_{1-7}\in \;\left(0.5;\right.0.7\rangle$—H (High);
- $K_{1-7}\in \;\left(0.7;\right.1\rangle$—VH (Very High).

When analysing Table 2, it can be seen that in the case of the prototype with *p* < 1, the values of the five criteria, *K*_1_, *K*_2_, *K*_3_, *K*_4_, *K*_7,_ are at the VL level, and the other two criteria, *K*_5_ and K_6,_ at the L level. At the same time, this prototype has the highest values of L_c1_ and L_c3_ and the lowest wear rate among the tested prototypes. For the prototype with *p* = 1, four of the examined criteria, *K*_1_, *K*_3_, *K*_4_, *K*_6,_ are at the VH level, the *K*_7_ criterion is at the H level, and *K*_5_ and *K*_2_ are at the L and VL levels, respectively. Along with the increase in the value of the above-mentioned criteria compared to the value of these criteria for the prototype with *p* < 1, a significant decrease in the value of the critical loads is observed by about 50%, as well as an almost 2.5-times increase in the wear rate.

In the case of the prototype with *p* > 1, the criteria *K*_3_, *K*_4_, *K*_6_ are also at the VH level, and the criteria *K*_1_ and *K*_7_ at the H level; however, the main difference from the prototype with *p* = 1 is the fact that the values of the *K*_3_ and *K*_4_ criteria take the extreme value equal to 1 and the *J*(*d_t_*,*p*) index takes the maximum value from the tested range. This means that in the case of this prototype, extreme and unfavorable effects deteriorating the anti-wear properties should be expected. Although, for the prototype with *p* > 1, the values of L_c1_ and L_c2_ are slightly higher compared to the prototype with *p* = 1, extensive buckling spallations are observed in the scratch test in this prototype. The obtained results clearly indicate that the prototypes with *p* > 1 are characterised by lower resistance to brittle cracking and delamination compared to the prototypes with *p* = 1 and *p* > 1. At the same time, prototypes with *p* > 1 are characterised by a much higher value of the J index than prototypes with *p* = 1 and *p* < 1, for which this index has the lowest values. It follows that the adopted index *J*(*d_t_*,*p*) can be regarded as a kind of measure of the coating resistance to the occurrence of so-called catastrophic failure modes. The obtained results also prove that using only the critical load L_c1_ as a measure of the quality of the coating adhesion to the substrate and its fracture toughness is ambiguous, because coatings with similar L_c1_ values may have different types of cracks with the same normal force value in the scratch test. 

## 4. Conclusions

The main aim of the conducted study was to select the optimal structure of the gradient coating in terms of its anti-wear properties, using an optimisation procedure proposed. Using the optimisation procedure with postulated criteria, an optimal FGM coating’s structure was proposed. The chosen prototypes (being the representatives of three main groups of solutions) were produced, and their properties were experimentally evaluated. 

For the needs of the optimal solution selection, the *J*(*d_t_*,*p*) quality index was developed, which facilitated the diversification of prototypes in terms of all criteria at the same time, creating subsets of solutions corresponding to the coatings with the best and worst possible anti-wear properties. Using the defined index, it was determined that optimal coatings should be characterised by:

- Exponent of the power transition function *p* < 1 (*J*(*d_t_*, *p*) *→ min*) and the thickness of the gradient layer *d_t_* at 80% of the total thickness of the coating;
- The asymmetric Lorentzian distribution of Young’s modulus and hardness (Figure 9), the maxima of which occur at a distance of about 20% *d_t_* from the adhesive layer interface;
- A strong decrease in hardness and Young’s modulus starting from the maximum, up to the top layer interface.

Based on the experimental tests (scratch tests, ball-on-disc tribological tests), it was found that the anti-wear properties of gradient coatings are related to the values of the adopted decision criteria. Namely, it has been shown that the prototypes with lower wear rates and higher critical loads are characterised by the lowest values of the *K*_1_–*K*_7_ criteria—the values of VL and L in the fuzzy-logic variable space.

Validation tests of the developed criteria can be extended to other Me–(C) coatings and will constitute an important level in the development of a universal and generally acceptable set of criteria and guidelines for the design of gradient coatings with enhanced anti-wear properties. As part of further research, it is planned to test the developed set of criteria in terms of stability and assessment of the impact of criteria weights on the individual anti-wear properties of other transition metal carbide coatings, e.g., TiC, VC, WC.

## Figures and Tables

**Table 1:** Values of *K*_1_–*K*_7_ criteria and $J=J\left(d_{t},p\right)$ for selected prototypes.

*p*	*d_t_*, µm	*K*_1_	*K*_2_	*K*_3_	*K*_4_	*K*_5_	*K*_6_	*K*_7_	*N*	*J*
0.25	2	0.019	0.003	0.068	0	0.193	0.232	0	7	0.044
1	1.75	0.853	0.064	0.778	0.760	0.115	0.820	0.622	2	0.93
3	2	0.557	0.051	1	1	0.405	0.799	0.597	1	1.864

**Table 2:** Summary of numerical simulations and experimental tests of selected prototypes.

*p*	*d*_*t*_, µm	*K*_1_	*K*_2_	*K*_3_	*K*_4_	*K*_5_	*K*_6_	*K*_7_	*J*	L_c1_, N	L_c3_, N	*W* 10^−7^ mm^3^/Nm	H, GPa
0.25	2.00	VL	VL	VL	VL	L	L	VL	0.05	23	60	1.08	21
1.00	1.75	VH	VL	VH	VH	L	VH	H	0.87	11	31	2.5	23
3.00	2.00	H	VL	VH	VH	M	VH	H	1.86	13	37	1.9	25

## Data Availability

Data sharing is not applicable to this article. Ongoing research project.
